# Aberrant Functional Connectivity between the Amygdala and the Temporal Pole in Drug-Free Generalized Anxiety Disorder

**DOI:** 10.3389/fnhum.2016.00549

**Published:** 2016-11-04

**Authors:** Wei Li, Huiru Cui, Zhipei Zhu, Li Kong, Qian Guo, Yikang Zhu, Qiang Hu, Lanlan Zhang, Hui Li, Qingwei Li, Jiangling Jiang, Jordan Meyers, Jianqi Li, Jijun Wang, Zhi Yang, Chunbo Li

**Affiliations:** ^1^Shanghai Key Laboratory of Psychotic Disorders, Shanghai Mental Health Center, Shanghai Jiao Tong University School of MedicineShanghai, China; ^2^College of Education, Shanghai Normal UniversityShanghai, China; ^3^Department of Psychology, Qiqihar Mental Health CenterQiqihar, China; ^4^Guangji Hospital of SuzhouSuzhou, China; ^5^Department of Psychiatry, Tongji Hospital of Tongji UniversityShanghai, China; ^6^Nathan S. Kline Institute for Psychiatric ResearchNew York, NY, USA; ^7^Shanghai Key Laboratory of Magnetic Resonance, Department of Physics, East China Normal UniversityShanghai, China; ^8^Key Laboratory for the Genetics of Developmental and Neuropsychiatric Disorders, Bio-X Institutes, Ministry of Education, Shanghai Jiao Tong UniversityShanghai, China; ^9^Brain Science and Technology Research Center, Shanghai Jiao Tong UniversityShanghai, China; ^10^CAS Key Laboratory of Behavioral Science and MRI Research Center, Institute of Psychology, Chinese Academy of SciencesBeijing, China

**Keywords:** amygdala, DLPFC, temporal pole, DMN, functional connectivity, generalized anxiety disorder

## Abstract

The amygdala and the dorsolateral prefrontal cortex (DLPFC) play important roles in “emotion dysregulation,” which has a profound impact on etiologic research of generalized anxiety disorder (GAD). The present study analyzed both eyes-open and eyes-closed resting state functional MRI (rs-fMRI) of 43 subjects (21 GAD patients with medicine free and 22 matched healthy controls). The amygdala and the DLPFC were defined as regions of interest (ROI) to analyze functional connectivity (FC) in GAD patients compared with healthy controls. The main findings revealed GAD patients had increased FC between the amygdala and the temporal pole compared to healthy controls, which was found in both eyes-open and eyes-closed rs-fMRI. And altered FC between the ROIs and brain regions that mainly belonged to the default mode network (DMN) were found. These findings suggest that the abnormal FC between the amygdala and the temporal pole may contribute to the pathophysiology of GAD, and provide insights into the current understanding of the emotion dysregulation of anxiety disorders.

## Introduction

Anxiety disorders are the most common of all mental disorders with 30% prevalence in the population, and they significantly contribute to the economic burden of disease (Andlin-Sobocki and Wittchen, 2005; Kessler et al., 2005; Bereza et al., 2009). Among anxiety disorders, generalized anxiety disorder (GAD) is the most common type (Roy-Byrne and Wagner, 2004; Lieb et al., 2005; Kroenke et al., 2007). GAD is characterized by excessive and continuous worry, anxiety, and apprehension. It may also produce distress and/or functional impairments.

Previous studies have argued that “emotional dysregulation,” the inability to control or regulate emotional responses, may be responsible for the development of GAD. This hypothesis is grounded in the observation that individuals with GAD concentrate their attention on threatening thoughts. This cognitive model has been widely adopted for understanding GAD (Mathews and MacLeod, 1985; Bar-Haim et al., 2007; Amir et al., 2009; Behar et al., 2009).

The dorsolateral prefrontal cortex (DLPFC), an important region for performing cognitive operations during the regulation of emotional responses, has been shown to play a key role in the pathophysiology of GAD (MacDonald et al., 2000; Miller and Cohen, 2001; Blasi et al., 2007; Meyer et al., 2011; Moon et al., 2015). Altered activation of the DLPFC in patients with GAD has been associated with emotional dysregulation and attention deficit. Functional MRI studies have reported increased activity of the DLPFC under affective stroop and emotion reappraisal tasks in patients with GAD (Ball et al., 2012; Blair et al., 2012). Additionally, functional abnormalities of the amygdala, known as the most prominent “fear-circuit” structure in the brain that plays a central role in automatic affective processing, have been found in most anxiety disorders (LeDoux, 2000; Anderson et al., 2003; Ohman, 2005; Etkin and Wager, 2007; Adolphs, 2008; Shin and Liberzon, 2010; Linares et al., 2012). The amygdala has also demonstrated responsibility for facilitating perceptual processing and bottom-up emotional control in individuals with GAD (LeDoux, 2000; Davis and Whalen, 2001; Phelps, 2006). Models of emotional regulation have therefore focused primarily on the DLPFC and the amygdala. Accordingly, we assume that individuals with GAD display aberrant FC seeded from the amygdala and the DLPFC compared to healthy controls.

Studies have shed less light on the relationship between the temporal cortex, especially the temporal pole, and GAD. Although the function of the temporal pole is not well understood, a damaged temporal pole can impair ability to use experiential knowledge and therefore may cause affective symptoms (Funnell, 2001). The temporal cortex binds complex, highly processed perceptual inputs to visceral emotional responses (Olson et al., 2007). The temporal pole is located at the end of the ventral visual stream and is strongly interconnected with the amygdala (Nakamura and Kubota, 1996; Stefanacci and Amaral, 2002). It integrates conceptual knowledge and meaning with semantic, visual and auditory information (Carlson et al., 2014), and influences emotions via top-down modulations (Pehrs et al., 2015). Studies have reported altered functional connectivity (FC) between the temporal pole and the amygdala in anxiety disorders (Aghajani et al., 2014; Modi et al., 2015). This finding has prompted us to explore the FC between the temporal pole and the amygdala in GAD patients. Therefore, one of our hypotheses is that altered FC between these two areas is attributed to the etiology of GAD.

Accordingly, the amygdala and the DLPFC will be defined as regions of interest (ROIs) to explore in GAD patients. Because the number of volumes in the eyes-open resting state fMRI (rs-fMRI) we collected was too small, we also analyzed the eyes-closed rs-fMRI using the same protocol to improve the reliability of our results.

## Materials and methods

### Participants

All participants received the Mini-International Neuropsychiatric Interview (MINI), Chinese version (Si et al., 2009). Twenty two GAD patients who met the criteria for DSM-IV (Association, 2000) and who were not found to have lifetime psychosis, substance dependence or severe somatic diseases were recruited from the psychological outpatient clinic at the Shanghai Mental Health Center. We excluded patients who had comorbid moods or other anxiety disorders. Twenty one healthy controls were recruited from local communities and Shanghai Jiao Tong University. Controls were matched for gender, age, education level, and did not meet DSM-IV criteria for lifetime mood, anxiety, psychotic, or substance dependence disorders. Forty three participants were enrolled in total for this study and according to the Edinburgh Inventory (Oldfield, 1971), all of them are right-handed adults free of psychotropic medications for at least 2 weeks before enrollment. The study was conducted between August 2011 and November 2012.

This study was approved by the Research Ethics Committee of Shanghai Mental Health Center, China (SMHC-IRB 201217). Written informed consent was acquired from every participant.

All participants were informed of the safety and eligibility criteria for fMRI scanning: no neurological conditions and no implanted ferrous metal. The Hamilton Rating Scale for Anxiety (HAMA) (Hamilton, 1959) and Hamilton Rating Scale for Depression (HAMD) (Hamilton, 1967) were administered to all participants on the day of scanning. Demographic and clinical characteristics of the 43 participants are shown in Table 1.

**Table 1:** **Demographic and clinical data**.

**Parameter**	**GAD**	**HC**	***p*-value**
	***n* = 21**	***n* = 22**	
Age (years)	39.90 ± 12.24	38.05 ± 10.32	0.593
Gender (M/F)	13/7	14/8	0.927
Education (years)	11.19 ± 3.31	12.50 ± 2.59	0.142
HAMA	18.6 ± 9.01	0.76 ± 0.94	0.000
HAMD	9.23 ± 5.10	0.86 ± 1.20	0.000

HAMA, Hamilton Anxiety Scale; HAMD, Hamilton Depression Scale;

*GAD, generalized anxiety disorder; HC: healthy control*.

### Image acquisition

Images were obtained using a Siemens Trio 3.0 Tesla MRI scanner (Siemens, Erlangen, Germany) with a standard 12-channel head coil. Restraining foam pads was used to reduce head motion and earplugs were used to reduce scanner noise. High-resolution T1-weighted anatomical images (repetition time (TR) = 1900 ms, echo time (TE) = 2.46 ms, flip angle = 9 degrees, 32 transverse slices, field of view (FOV) = 240 × 240 mm, matrix = 256 × 256, slice thickness = 1 mm) were acquired using a magnetization prepared rapid gradient-echo sequence. Resting-state functional MRI data were acquired using a single-shot, gradient-recalled echo planar imaging sequence (TR = 2000 ms, TE = 25 ms, flip angle = 90 degrees). 32 transverse slices (FOV = 240 × 240 mm, matrix = 64 × 64, slice thickness = 5 mm) resulting in a total of 80/157 volumes and a scan time of 164/314 s, respectively, in eyes-open and eyes-closed rs-fMRI. During the scan, participants were instructed to stay aware. After the scan, the technicians would check the quality of structural images. If any abnormalities were found in the images, participants were re-scanned.

### Data processing and analysis

#### Demographic and clinical data analysis

Using Statistical Product and Service Solutions software 17.0 (SPSS, Inc., Chicago, Illinois), we conducted analysis of age, gender, years of education, HAMA, and HAMD. Independent sample *t*-tests for continuous variables and chi-square tests for categorical variables were used.

#### Resting-state fMRI analysis

The Data Processing Assistant for Resting-State fMRI 2.0 (DPARSFA2.0, http://restfmri.net/forum/) (Chao-Gan and Yu-Feng, 2010), which works with the Statistical Parametric Mapping Software (SPM8, http://www.fil.ion.ucl.ac.uk/spm) (Friston et al., 1994) was used to analyze the rs-fMRI data. Preprocessing was completed in 7 steps: (1) Convert DICOM data to NIFTI format and remove first 10 time points of the image; (2) Slice timing correction and realignment of image; (3) Parallel movements in any direction >2.5 mm, or rotary movements >2.5 degree were excluded and subjects using a threshold of frame-wise displacement >0.5 mm (Power et al., 2012) were also excluded; (4) Spatial normalization to the standard Montreal Neurological Institute (MNI) echo-planar imaging template and the resampled voxel size was 3 × 3 × 3 mm; (5) Conduct Friston 24-parameter correction (Yan et al., 2013) to minimize the effect of head motion; (6) Smoothing with a Gaussian kernel of 8-mm full-width at half-maximum (FWHM); (7) After linear detrending, the functional data was band-pass filtered (pass frequence band: 0.01–0.1 Hz) to reduce the effects of low-frequency drift as well as high-frequency respiratory and cardiac noise (Biswal et al., 1995).

According to previous studies (Cieslik et al., 2013; Comte et al., 2014; Cui et al., 2016), the bilateral amygdala (MNI: 32,−2,−26;−28, 4,−22) and the bilateral DLPFC (MNI: 30, 43, 23;−51, 27, 30) were defined as ROIs. The peak voxel of each ROI and a 6 mm-radius sphere were selected to proceed with the FC analysis. The Pearson correlation coefficients were calculated between the ROI and the other voxels of the whole brain. Fisher's r-to-z transformation was used to convert correlation coefficients into z-scores so that the correlation coefficient would improve the normality of the data (Hampson et al., 2002; Chao-Gan and Yu-Feng, 2010; Song et al., 2011), and generate FC maps. Voxel-wise two-sample *t*-tests were conducted to compare group differences between GAD patients and controls. Spearman correlation analysis was performed in GAD patients to investigate the correlation between FC and disease severity (HAMA score). Both *t*-tests and correlation analysis were conducted under the BrainMask_61^*^73^*^61. As correction for multiple comparisons, a corrected threshold of *p* < 0.05 (two-tailed) was derived from a combined threshold of *p* < 0.005 for individual voxel with a cluster size >53 voxels. These threshold were determined using the 3dFWHM and 3dClustSim program in AFNI software (https://afni.nimh.nih.gov/afni, parameters: single voxel *p* < 0.005, 2000 Monte Carlo iterations, estimated FWHM = 9.5 mm, the BrainMask_61^*^73^*^61 was used as mask in estimation of smoothness and correction). Additionally, age, years of education, HAMD score and intracranial volume (ICV) were modeled as covariates. The analyses above were conducted in both eyes-open and eyes-closed rs-fMRI.

In order to improve the reliability of these results, we calculated mean frame-wise displacement for each group and conducted *t*-tests between matched groups in SPSS.

## Results

### Demographic and clinical characteristics

Compared with the healthy control group, the HAMA scores and HAMD scores were significantly different in GAD patients (*P* < 0.05). Except that, there is no significant difference between GAD patients and healthy controls.

### Resting-state fMRI results

#### Functional connectivity

Compared with healthy controls, GAD patients showed increased FC between the left amygdala and the temporal pole both in eyes-open and eyes-closed rs-fMRI (*P* < 0.005 to define cluster, AlphaSim correction, cluster size >53 voxels, overall *p* < 0.05).

**Figure 1:**
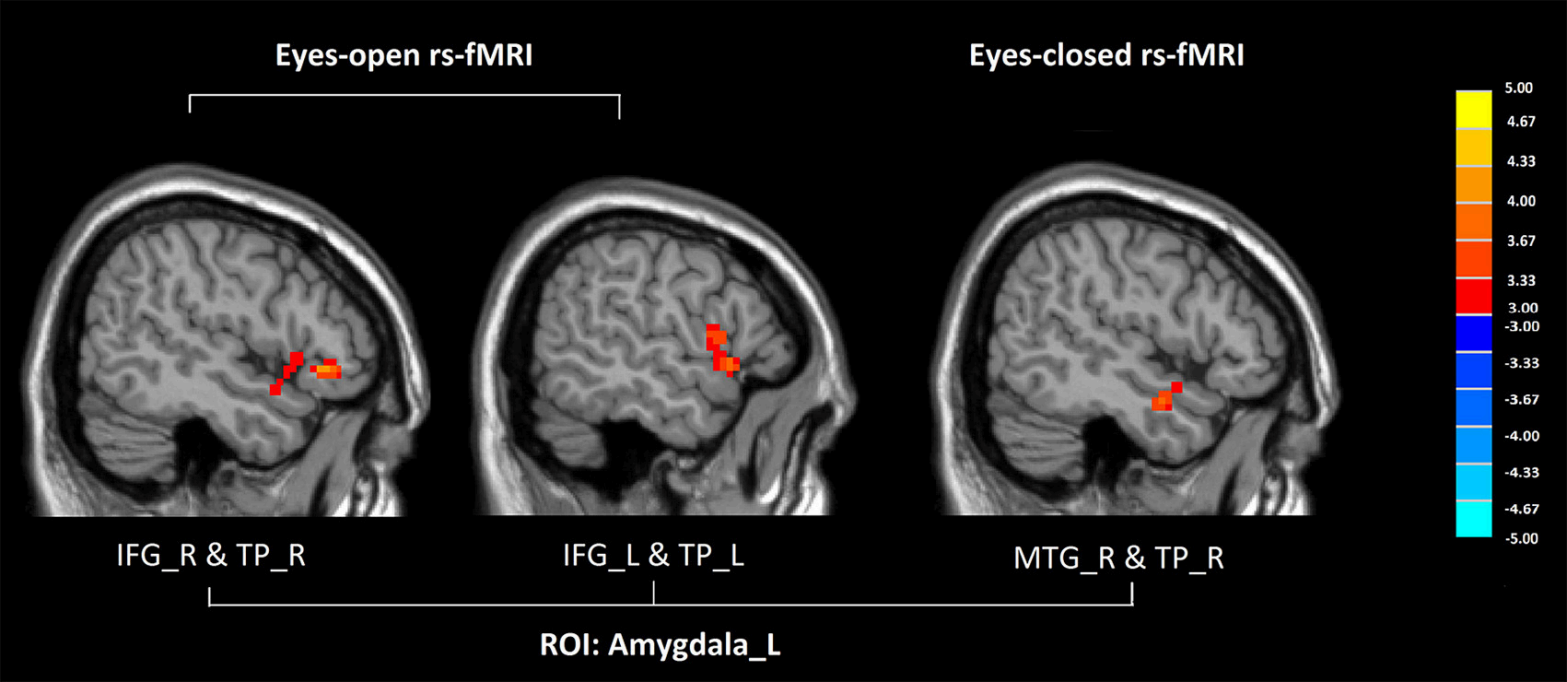
**Altered functional connectivity seeded from the left amygdala in GAD, compared with HC (***P*** < 0.005 to define cluster, AlphaSim correction, cluster size >53 voxels, overall ***p*** < 0.05)**. Hot colors indicate increased functional connectivity in GAD compared with HC. (GAD, generalized anxiety disorder; HC, healthy control; rs-fMRI, resting state fMRI; IFG, inferior frontal gyrus; TP, temporal gyrus; MTG, middle temporal gyrus; L, left; R, right).

In eyes-open rs-fMRI, there was increased connectivity between the left amygdala and the inferior frontal gyrus in the GAD group compared with the control group (*P* < 0.005 to define cluster, AlphaSim correction, cluster size >53 voxels, overall *p* < 0.05). (Figure 1, Table 2) There was no significant abnormal FC between GAD subjects and controls seeded from the DLPFC.

**Table 2:** **Alterations in FC seeded from the amygdala and DLPFC between GAD and HC**.

**Brain regions**	**BA**	**MNI coordinates**			**Voxel**	**Peak *t*-value**
		***X***	***Y***	***Z***		
**EYES-OPEN rs-fMRI**						
**ROI: AMYGDALA_L**						
Inferior frontal gyrus_R	47, 22, 38	57	36	−6	76	4.40
**Temporal pole_R**						
Inferior frontal gyrus_L	22, 44, 47	−57	12	−3	58	3.93
Temporal pole_L						
**EYES-CLOSED rs-fMRI**						
**ROI: AMYGDALA_L**						
Middle temporal gyrus_R	21, 38	48	−6	−24	54	4.12
**Temporal pole_R**						
**ROI: DLPFC_R**						
Medial prefrontal cortex_L/R	11, 32, 25	6	36	−9	867	−6.16
Dorsal anterior cingulate cortex_L/R						
Middle temporal gyrus_R	21	60	3	−21	95	−4.84
Precuneus_L						
Calcarine sulcus_L	30, 29, 23	6	−51	0	412	−4.93
Cerebellar vermis						
Angular gyrus_R	39	42	−63	21	74	−4.37
**ROI: DLPFC_L**						
Precuneus_L	29, 30	−21	−48	0	258	−4.14
Lingual gyrus_L						
Calcarine sulcus_L						
Cerebellar vermis						

*R, right; L, left; BA, Brodmann's area; MNI, Montreal Neurological Institute; GAD, generalized anxiety disorder; HC, healthy control; FC, functional connectivity; DLPFC, dorsolateral prefrontal cortex; ROI, region of interest*.

**Figure 2:**
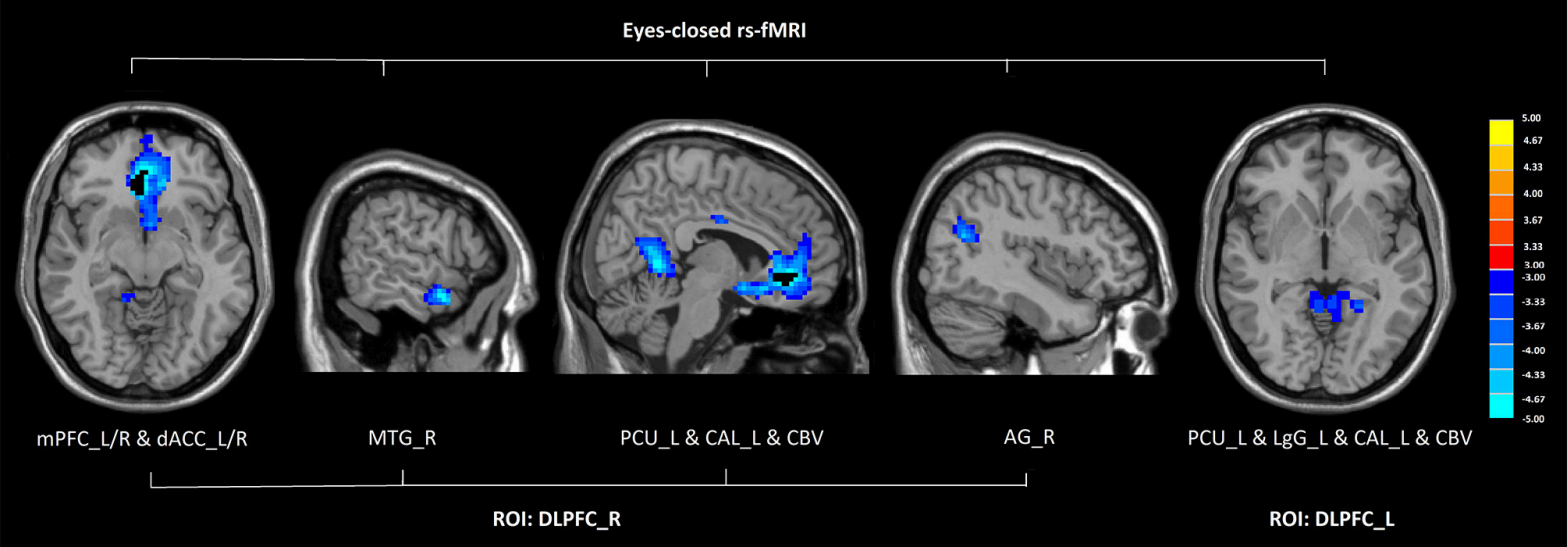
**Altered functional connectivity seeded from the bilateral DLPFC in GAD, compared with HC (***P*** < 0.005 to define cluster, AlphaSim correction, cluster size >53 voxels, overall ***p*** < 0.05)**. Hot and cold colors indicate increased and decreased FC in GAD compared with HC. (DLPFC, dorsolateral prefrontal cortex; GAD, generalized anxiety disorder; HC, healthy control; rs-fMRI, resting state fMRI; mPFC, medial prefrontal cortex; dACC, dorsal anterior cingulate cortex; MTG, middle temporal gyrus; PCU, precuneus; CAL, calcarine sulcus; CBV, cerebellar vermis; AG, angular gyrus; LgG, lingual gyrus; L, left; R, right).

In eyes-closed rs-fMRI, we found increased FC in the left amygdala with the middle temporal gyrus (*P* < 0.005 to define cluster, AlphaSim correction, cluster size >53 voxels, overall *p* < 0.05). Decreased FC between the left DLPFC and the precuneus, the lingual gyrus, the calcarine sulcus, and the cerebellar vermis was detected in GAD subjects compared with healthy controls (*P* < 0.005 to define cluster, AlphaSim correction, cluster size >53 voxels, overall *p* < 0.05). There was decreased connectivity between the right DLPFC and the medial prefrontal cortex (mPFC), the dorsal anterior cingulate cortex (dACC), the middle temporal gyrus, the angular gyrus, the precuneus, the calcarine sulcus, and the cerebellar vermis in GAD subjects compared with healthy controls (*P* < 0.005 to define cluster, AlphaSim correction, cluster size >53 voxels, overall *p* < 0.05). (Figure 2, Table 2).

#### Correlation analysis of FC and illness severity

In eyes-open rs-fMRI, the HAMA score had a significant negative correlation with the FC between the left amygdala and the superior frontal gyrus in GAD subjects (*P* < 0.005 to define cluster, AlphaSim correction, cluster size >53 voxels, overall *p* < 0.05). The FC between the right amygdala and the fusiform gyrus, the superior/middle occipital gyrus, and the cerebellum was positively correlated to the HAMA score in GAD subjects (*P* < 0.005 to define cluster, AlphaSim correction, cluster size >53 voxels, overall *p* < 0.05). We found that the HAMA score was positively correlated with the FC between the DLPFC and the inferior frontal gyrus, the supplementary motor area (SMA), and the cerebellum in GAD subjects (*P* < 0.005 to define cluster, AlphaSim correction, cluster size >53 voxels, overall *p* < 0.05). (Table 3, Supplement Figure 1)

**Table 3:** **Correlation between altered functional connectivity and HAMA scores for GAD patients**.

**Brain regions**	**BA**	**MNI coordinates**			**Voxel**	**Rho**
		***X***	***Y***	***Z***		
**EYES-OPEN rs-fMRI**						
**ROI: AMYGDALA_R**						
Fusiform gyrus_L	37, 19	−24	−42	−18	66	0.77
Cerebellar vermis	NA	6	−48	−9	100	0.82
Cerebellum_L						
Superior occipital gyrus_R	19	24	−78	36	71	0.81
Middle occipital gyrus_R						
**ROI: AMYGDALA_L**						
Superior frontal gyrus_L	9	−3	36	33	67	−0.79
**ROI: DLPFC_R**						
Cerebellum_R	NA	12	−48	−60	76	0.81
Supplementary motor area_L	6	−15	3	75	72	0.85
**ROI: DLPFC_L**						
Cerebellum_R	NA	33	−48	−51	78	0.83
Inferior frontal gyrus_L	38, 47	−51	24	6	119	0.84
**EYES-CLOSED rs-fMRI**						
**ROI: DLPFC_R**						
Cerebellum_R	19	30	−51	−18	70	0.82
Fusiform gyrus_R						
Fusiform gyrus_L	37, 19	−24	−51	−12	80	0.82
Lingual gyrus_L						
Inferior occipital gyrus_L	19	−48	−72	−9	74	0.86
Middle occipital gyrus_R	19	42	−78	9	69	0.85
Cuneus_ R	19, 18	6	−81	39	125	0.81

*R, right; L, left; BA, Brodmann's area; MNI, Montreal Neurological Institute; GAD, generalized anxiety disorder; HC, healthy control; DLPFC, dorsolateral prefrontal cortex; HAMA, Hamilton Anxiety Scale; ROI, region of interest*.

In eyes-closed rs-fMRI, the HAMA score had a significant positive correlation with the FC between the right DLPFC and the lingual gyrus, the cuneus, the fusiform gyrus, the inferior/middle occipital gyrus, and the cerebellum in GAD subjects (*P* < 0.005 to define cluster, AlphaSim correction, cluster size >53 voxels, overall *p* < 0.05). (Table 3, Supplement Figure 1).

Additionally, both in eyes-open and eyes-closed rs-fMRI, we did not find a significant difference in frame-wise displacement between GAD patients and healthy controls [*p* = 0.926 (eyes-open), 0.271 (eyes-closed)].

## Discussion

In this study, we conducted analysis to characterize alterations in FC that may show the pathological basis of GAD using both the eyes-open and the eyes-closed rs-fMRI. While exploring FC seeded from the amygdala and the DLPFC, we found: (1) Patients with GAD showed increased FC between the left amygdala and the temporal pole compared with health controls. (2) In both eyes-open and eyes-closed conditions, the brain regions showed altered FC with amygdala/DLPFC were primarily from the default mode network (DMN).

Increased FC between the amygdala and the temporal pole was detected by both eyes-open and eyes-closed rs-fMRI. This finding may illustrate that the altered FC may contribute to the etiology of GAD. Previous findings indicate that the temporal pole plays a role in both social and emotional processing, including specific recognition, theory of mind (Wong and Gallate, 2012), memory (Damasio et al., 1996), and encodes similarity relations among different concepts (Patterson et al., 2007). It is also thought to be involved in access to knowledge during “mentalizing,” which refers to the attribution of intentions and other mental states (Frith and Frith, 2003). The amygdala is one of the most investigated structures of the brain, especially in the context of emotional processing. The amygdala is marked as the most prominent “fear-circuit” structure, and hyperactivation of the amygdala is found in most anxiety disorders (Etkin and Wager, 2007; Shin and Liberzon, 2010; Linares et al., 2012). And it has been studied extensively within the context of fear conditioning and extinction as key processes for the pathophysiology of anxiety disorders. The FC between the amygdala and the temporal pole may reflect integration of emotional regulation with knowledge during stimuli perception and mentalizing in healthy subjects. This neural process is presumably disrupted in GAD, which is consistent with observations of deficits in socio-emotional behaviors (Aghajani et al., 2014). The increased connectivity between the temporal pole and the amygdala in GAD patients may be responsible for why stimuli more easily evoke anxiety in GAD patients.

The default mode network (DMN) is defined as the set of regions in the brain that are consistently more activated during resting condition than other brain networks (Fox and Raichle, 2007). It is often described as a unitary, homogeneous system that is largely involved in the integration of autobiographical memories and in self-monitoring, in the retrieval and manipulation of past events in an effort to solve problems and develop future plans, and in emotion regulation (Greicius et al., 2003). When a task requires attention, however, the activation of such network is suppressed. Deficits in DMN suppression are reported in several mental illnesses, notably anxiety disorders (Anticevic et al., 2012). Our results showed altered FC between the amygdala and several regions of the brain including the temporal pole, the middle temporal gyrus, which belong to the DMN, in the GAD group compared with the control group. Altered FC was also found between the DLPFC and brain regions of the DMN, such as the mPFC, the angular gyrus, and the precuneus in the GAD group.

The middle temporal gyrus is regarded as an important brain structure in the integration of memory, audiovisual association, object-recognition and visual perception (Li et al., 2013; Shao et al., 2013). The middle temporal gyrus was found to have increased FC between the amygdala. This finding may reflect an increased predisposition for inaccurate interpretation of stimuli (Pannekoek et al., 2013). However, the FC between the DLPFC and the middle temporal gyrus, and the mPFC was decreased in GAD patients compared with healthy controls. The DLPFC is involved in the function of working memory, executive functions, emotion regulation, subjective feelings, and self-awareness (Craig, 2002; Critchley et al., 2004). The mPFC is widely known to be crucial for emotion regulation, especially for controlling negative emotional responses (Etkin et al., 2009). The angular gyrus, which is also a part of the DMN, has been implicated in affective regulation associated with empathic response, anxiety, and mood (Leung et al., 2013). The precuneus is implicated in episodic memory, visuospatial processing, self-reflection and aspects of consciousness (Fox et al., 2015; Hannawi et al., 2015; Kwok and Macaluso, 2015). The decreased FC between the DLPFC and the brain regions in the DMN may explain uncontrolled emotional regulation in GAD patients. This finding was consistent with our previous study and the models of “bottom-up” and “top-down” emotional processing (Phillips et al., 2003, 2008; Ochsner and Gross, 2005; Phan et al., 2005; Goldin et al., 2008; Cui et al., 2016).

The lingual gyrus, the cuneus, and the fusiform gyrus all belong to the visual network (VN). Functional abnormalities of these regions reflect excessive vigilance as a hallmark of anxiety disorders. The VN is associated with pathological memories and planning a response to potentially threatening stimuli (Bremner et al., 1999). The dACC, a part of the salience network (SN), plays a central role in detecting emotional salience and triggering cognitive control via FC with the DLPFC (Sridharan et al., 2008; Bressler and Menon, 2010). Both the DLPFC and the dorsal ACC are implicated in emotional regulation circuits (Bush et al., 2000; Brühl et al., 2014). Therefore, the decreased FC between the DLPFC and the VN/SN may be related to the loss of emotional regulation from the DLPFC in GAD patients. The cerebellum is linked with the cerebrum, brainstem, and spinal cord through efferent and afferent fibers, and the cerebellar vermis is connected to the amygdala anatomically in animals (De Bellis et al., 2002). Recently, more and more studies have reported the cerebellum is functionally related to expressing fear and processing fear memory (Supple et al., 1987; Sacchetti et al., 2005). Cerebellar cognitive affective syndrome was observed in patients with cerebellar damage (Stoodley, 2012). The DLPFC is involved in executive control (Habas et al., 2009; O'Reilly et al., 2010; Yeo et al., 2011), so the connectivity between the cerebellum and the DLPFC may mediate anxiety (Caulfield et al., 2016). And it supports our finding that the decreased FC between the DLPFC and the cerebellum in GAD patients compared with healthy controls.

Regarding the underlying mechanism, the temporal lobe, especially the temporal pole, may be the emphasis for future treatment of GAD. Although there some studies reported the effect of transcranial direct current stimulation (tDSC) and repetitive transcranial magnetic stimulation (rTMS) in curing GAD, the outcomes are various. According to our findings, we prefer the temporal pole as the stimulated target. By decreasing the related abnormal FC may remit anxiety in GAD patients.

## Conclusion and limitations

In conclusion, our study found altered FC seeded from the amygdala and the DLPFC in GAD patients using eyes-open and eyes-closed rs-fMRI. We found that the increased FC between the amygdala and the temporal pole may be underlying the neural pathophysiology of GAD. We hope these findings will shed light on the current understanding of GAD and on advanced therapeutic interventions.

A limitation in this study is that we were only able to acquire 164 s in the eyes-open resting state fMRI data and the reliability of FC analysis under this condition is limited (Shehzad et al., 2009; Thomason et al., 2011; Braun et al., 2012; Li et al., 2012). Nonetheless, the consistency between eyes-open and eyes-closed conditions alleviates this concern and provides support for our conclusion. Additionally, as fMRI data was acquired using the parameters TR = 2s, slices = 30, band pass filtering in the range 0.01 < f < 0.1 Hz, cardiac and respiratory fluctuations may still reduce the specificity of low frequency fluctuations to functional connected regions (Lowe et al., 1998). Future research will focus on MRI follow-up and will explore changes in neuroimaging of GAD.

## Author contributions

WL: manuscript, data gathering and analysis. HC, ZZ, QH, LZ, JM, and HL: manuscript and data gathering. LK, JJ, QG, and YZ: manuscript and analysis. JL and QL: data gathering and quality control. JW and ZY: manuscript, administration, and editing. CL: research plan development, manuscript, administration, and editing.

## Funding

Funding for this study was provided by the National Natural Science Foundation of China (81071098, 81270023, 81571756)(to CL and ZY), Shanghai Health System Leadership in Health Research Program (XBR2011005) (to CL), Shanghai Health Bureau Project (2013SY003) (to JW), the Science and Technology Commission of Shanghai Municipality (13dz2260500, 15411950201, 14411961400, 20154Y0080) (to CL, ZZ, and JW), Shanghai Clinical Center for Mental Disorders (2014), National Key Clinical Disciplines at Shanghai Mental Health Center (Office of Medical Affairs, Ministry of Health, 2011-873; OMA-MH, 2011-873)(to HC), Beijing Nova Program for Science and Technology (XXJH2015B079)(to ZY), Pfizer Investigator Initiation Research Fund (WI173560)(to CL) and Chinese Government Scholarship (CSC NO.201606230115)(to WL).

### Conflict of interest statement

The authors declare that the research was conducted in the absence of any commercial or financial relationships that could be construed as a potential conflict of interest.
