# Supplementary material for: Aberrant Functional Connectivity between the Amygdala and the Temporal Pole in Drug-Free Generalized Anxiety Disorder
Source: Front Hum Neurosci. 2016 Nov 4;10:549. doi: 10.3389/fnhum.2016.00549 (PMC5095112; doi:10.3389/fnhum.2016.00549)
Supplement: Supplementary file 1 [file Image1.PDF]

Supplement

Figure 1. Correlation between altered functional connectivity and HAMA scores for GAD patients (P<0.005 to define cluster, AlphaSim correction, cluster size >53 voxels, overall p<0.05).

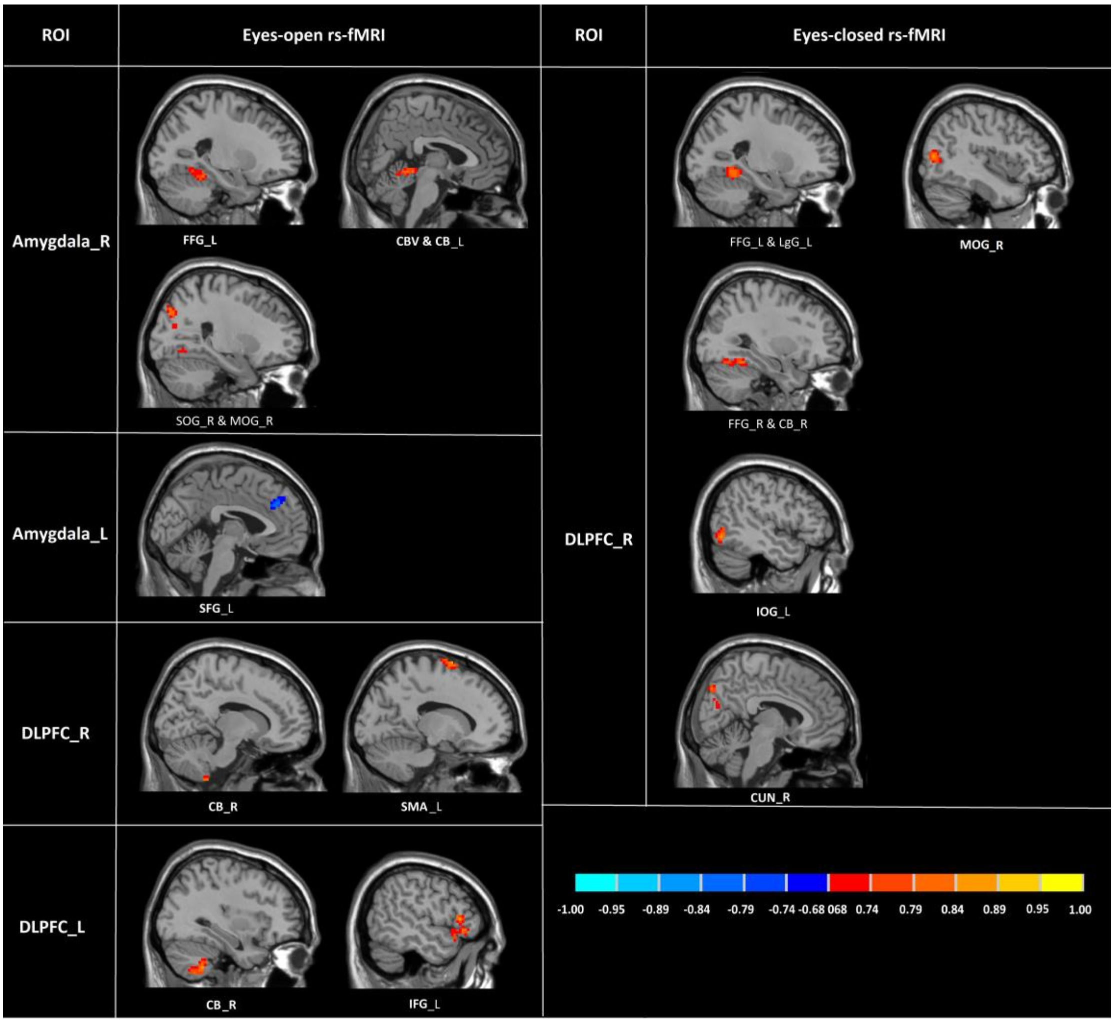

(GAD: generalized anxiety disorder; rs-fMRI: resting state fMRI; ROI: region of interest; FFG: Fusiform gyrus; CBV: cerebellar vermis; CB: cerebellum; SOG: superior occipital gyrus; MOG: middle occipital gyrus; IOG: inferior occipital gyrus; SFG: superior frontal gyrus; IFG: inferior frontal gyrus; SMA: supplementary motor area; DLPFC: dorsolateral prefrontal cortex; LgG: lingual gyrus; CUS: cuneus; L: left; R: right)
